# *C*. *elegans* SMA-10 regulates BMP receptor trafficking

**DOI:** 10.1371/journal.pone.0180681

**Published:** 2017-07-13

**Authors:** Ryan J. Gleason, Mehul Vora, Ying Li, Nanci S. Kane, Kelvin Liao, Richard W. Padgett

**Affiliations:** Waksman Institute, Department of Molecular Biology and Biochemistry, Cancer Institute of New Jersey, Rutgers University, Piscataway, New Jersey, United States of America; University of North Carolina at Chapel Hill, UNITED STATES

## Abstract

Signal transduction of the conserved transforming growth factor-β (TGFβ) family signaling pathway functions through two distinct serine/threonine transmembrane receptors, the type I and type II receptors. Endocytosis orchestrates the assembly of signaling complexes by coordinating the entry of receptors with their downstream signaling mediators. Recently, we showed that the *C*. *elegans* type I bone morphogenetic protein (BMP) receptor SMA-6, part of the TGFβ family, is recycled through the retromer complex while the type II receptor, DAF-4 is recycled in a retromer-independent, ARF-6 dependent manner. From genetic screens in *C*. *elegans* aimed at identifying new modifiers of BMP signaling, we reported on SMA-10, a conserved LRIG (leucine-rich and immunoglobulin-like domains) transmembrane protein. It is a positive regulator of BMP signaling that binds to the SMA-6 receptor. Here we show that the loss of *sma-10* leads to aberrant endocytic trafficking of SMA-6, resulting in its accumulation in distinct intracellular endosomes including the early endosome, multivesicular bodies (MVB), and the late endosome with a reduction in signaling strength. Our studies show that trafficking defects caused by the loss of *sma-10* are not universal, but affect only a limited set of receptors. Likewise, in *Drosophila*, we find that the fly homolog of *sma-10*, *lambik* (*lbk*), reduces signaling strength of the BMP pathway, consistent with its function in *C*. *elegans* and suggesting evolutionary conservation of function. Loss of *sma-10* results in reduced ubiquitination of the type I receptor SMA-6, suggesting a possible mechanism for its regulation of BMP signaling.

## Introduction

The TGFβ family comprises a large family of protein ligands that is involved in many developmental decisions and is often associated with some diseases and cancers [1–4]. It is an ancient signaling family not present in yeast or plants, but is found in the most simpler animals such as sponges and trichoplax [5–7]. The ligands form a continuum of homology but can be roughly grouped into the TGFβ, the BMP, and the activin subfamilies [8, 9]. *C*. *elegans* has two complete signaling pathways, the Small and Male tail abnormal (Sma/Mab) pathway and the dauer pathway [10–12]. The Sma/Mab pathway, which is BMP-like, controls body size by regulating cell size rather than cell number, as well as male tail development, mesoderm development, and some aspects of innate immunity [10–13].

Unlike most tyrosine kinase signaling pathways, BMP signals through two related serine-threonine kinase transmembrane receptors [1, 9]. For signaling to occur, both receptors are required at the cell surface to physically interact with each other and their ligands. This interaction results in the phosphorylation of a downstream receptor activated-Smad (R-Smad) protein. This phosphorylation event triggers the binding of a common mediator Smad (Co-Smad) to form a heteromeric complex between R-Smad proteins and the Co-Smad. These Smad complexes accumulate in the nucleus where they can bind additional co-factors to activate or repress transcription.

After signaling, most transmembrane receptors are either recycled for new rounds of signaling or are degraded by the lysosome [14–19]. Once receptors enter early endosomes, they are either sorted into recycling pathways that will return the molecule to the plasma membrane for another round of signaling or are sorted into a degradative pathway [14, 16, 17, 20, 21]. This bifurcation represents an important point of regulation for signaling pathways, although little is known about the regulation of this post-endocytic process.

In the case of the BMP receptors, we have shown that the type I BMP receptor in *C*. *elegans*, SMA-6, is recycled through the retromer, whereas the type II receptor DAF-4 is recycled via a separate and distinct recycling pathway regulated by ARF-6 [22]. Two other studies indicate that the mammalian type I BMP receptor may also recycle through the retromer, suggesting this is a conserved mechanism for BMP type I receptors [23, 24].

Distinct sorting pathways may be required for BMP receptors, given the unique property that signaling requires two related receptors. Physically separating the two BMP receptors into molecularly distinct sorting complexes may be necessary to separate the type II receptor from the type I receptor in order to reduce signaling. Whether signaling has ceased by the time the receptors have separated is not known, but at minimum, separation of the receptors into distinct recycling pathways provides another avenue of BMP regulation.

Understanding how BMP signaling components are regulated is of considerable interest. Since BMP is a potent growth factor, cells have devised many different mechanisms to control its activity, such as N-glycosylation, ubiquitylation, sumolation, neddylation, and methylation [25, 26]. Previously, we reported on *sma-10*, which encodes a member of the LRIG family [27]. LRIG members have a characteristic set of 15 leucine-rich repeats and three immunoglobulin repeats in the ectodomain, juxtaposed to a transmembrane domain and a cytoplasmic domain [27–29]. Leucine-rich and immunoglobulin domains generally participate in protein-protein interactions [30]. Biochemical analysis of SMA-10 shows it binds to both the type I and type II receptors, SMA-6 and DAF-4, respectively [27]. Likewise, the human LRIG1 binds to the human type I and type II receptors, but not to the BMP ligands [27]. The three members of the vertebrate LRIG family (Lrig1/2/3) and the single *Drosophila* member, *lbk*, show only modest homology to each other in their ~300 amino acid cytoplasmic domains [27–29] in contrast to their more highly conserved extracellular domains. The *C*. *elegans* ortholog has a very short cytoplasmic domain, further highlighting the high divergence in this domain [27]. Expression of the human LRIG1, LRIG2, and LRIG3 paralogs are widespread in vertebrates and show some differential regulation in human tissues. All three have been characterized as tumor suppressors and/or proto-oncogenes [28, 31], consistent with their regulation of signaling pathways.

In this report, we address three questions: 1) where does SMA-10 act to regulate receptor trafficking, 2) is the effect of SMA-10 on BMP function conserved in other organisms, and 3) is SMA-10 specific for BMP signaling. In this work, we show that mutations in *sma-10* alter the intracellular trafficking of the type I (SMA-6) receptor in *C*. *elegans*. In *sma-10* mutants, the receptors enter the cell, but accumulate intracellularly after entry in distinct endocytic compartments. This receptor accumulation results in minimal BMP signaling. Ubiquitination of the BMP receptors is well documented for proper trafficking and signaling [32–34], but how that process is regulated is not well understood. We show that mutations in *sma-10* reduce the ubiquitination of the type I receptor, leading to mis-trafficking of the receptor and a decrease in signaling.

## Methods

### General methods and strains

All *C*. *elegans* strains are derived from the Bristol strain N2 [35] and were grown at 20°C on standard nematode growth media plates seeded with OP50 *E*. *coli*. Worm cultures, genetic crosses, and other *C*. *elegans* husbandry were performed according to standard protocols [35]. A complete list of strains used in this study can be found in S1 Table. RNAi was performed using the feeding method [36]. Feeding constructs were obtained from an RNAi library [37] or from cloned genomic DNA fragments. Larval stage L4 animals were treated for 24h and imaged as young adults.

### Plasmids and transgenic lines

Expression of TagRFP- and GFP- tagged versions of SMA-10 in the worm intestine was driven with the intestinal-specific promoter p*Vha-6* [22]. *C*. *elegans sma-10* genomic DNA, lacking the terminal stop codon, was cloned into the Gateway^™^ entry vector pDONR 221 (Invitrogen), and subsequently transferred into expression vectors by recombination cloning to generate C-terminal fusions. Microparticle bombardment of *unc-119(ed3)* mutant animals [38–40] was done to obtain low-copy integrated transgenic lines.

Generation of truncation mutants for the *sma-10(wk88)* body size rescue was achieved using mutagenesis using the Q5 mutagenesis kit (Life technologies) as per manufacturer’s instructions. A GFP-tagged full length *sma-10* expressed from a hypodermal promoter (*elt-3*) was mutagenized to remove the cytoplasmic tail (ΔCyto) using the forward primer 5’- TAGCATTCGTAGAATTCC -3’ and reverse primer 5’- AATGCAAATTGAAGTGATAACTC -3’. The linearized PCR products were then subjected to the KLD reaction to circularize products and plasmids were confirmed by sequence.

### Microscopy and image analysis

Live worms were mounted on 2% (wt/vol) agarose pads with tetramisole. Using argon 488-nm excitation, we used the spectral profile function of the Leica SP5 confocal microscope to establish a spectral profile of the intestinal autofluorescence to separate it from the experimentally determined GFP spectrum. The worm intestine consists of 20 individual epithelial cells with distinct apical, lateral, and basal regions, positioned as bilaterally symmetric pairs to form a long tube around the lumen. The focal planes captured in this study are designated as the Top plane, which captures the top of the intestinal tube (basolateral surface), and the Middle plane, which captures the midsagittal cross section of the apical and basolateral surfaces.

Quantification of images was performed using the open-source Fiji software [41]. Within any set of comparable images, the image capture, scaling conditions and threshold values were identical for all images within a given experiment. For each experiment, at least six animals were analyzed with three randomly selected regions-of-interest (ROI) per animal. All the images were taken from the same cells of the intestine (either the second or third pair of cells relative to the anterior end of the intestine). Quantification of the fluorescence in each ROI was performed. Colocalization images were performed on L4 staged animals, using a confocal microscope equipped with the confocal imager (CARV II; BD Biosciences). Colocalization analysis was conducted using the Costes method to establish a threshold in Fiji software [41] which also outputs the Mander’s and Pearson’s correlation values.

Plasmid constructs expressing full length *sma-10 [sma-10(+)*,red] and *sma-10* lacking the cytoplasmic tail [*sma-10(Δcyto)*] from a hypodermal-specific promoter *elt-3* were injected into the *sma-10(wk88)* mutant background. Transformed animals were assayed for body size at the L4 stage+ 24 hr. Transformants were selected based on presence of GFP in the pharynx and imaged at 10X magnification using standard epifluorescent microscopes. Fiji software [41] was then used to measure body size from tip of the mouth (anterior) to the tip of the tail (posterior) using the Segmented Line tool.

### Protein expression and blots

Worms were synchronized by alkaline bleaching and grown on standard NGM plates until young adult stage (L4 + 24 hours). Animals were collected and washed in 5 ml M9 (three times at 1500 rpm). After the final wash, animals were resuspended in 750 μl lysis buffer (50 mm HEPES, 150 mm NaCl, 0.5 mM EDTA supplemented with protease inhibitor tablets (Roche Scientific Ltd). Approximately 500 μl of glass beads (#G8772-500G, Sigma Aldrich) were added and bead beating was carried out for 30 seconds at 4°C with a one minute incubation on ice between each of two bead beatings. Crude lysate was centrifuged at 18,000 rpm for 10 minutes at 4°C and the supernatant was transferred to a fresh pre-cooled microcentrifuge tube and centrifuged again. Supernatant was transferred to a pre-cooled microcentrifuge tube. Protein concentration was determined using the Bradford assay (500–0006, Bio-rad), and 50 μg of protein was mixed with 4x protein sample buffer (Amresco Inc.) and boiled for 10 minutes. These input samples were then stored at –80°C until used for immunoblotting. 20 μl of resuspended Agarose A/G beads (Santa Cruz Biotech) were added to the remaining samples and incubated on a rocker at 4°C for 1 hour followed by a centrifugation at 1500 rpm for 5 minutes at 4°C. Supernatant was collected and incubated with mouse anti-GFP (Roche) for 1–4 hours at 4°C on a rocker platform. 20 μl of agarose A/G beads (Santa Cruz Biotechnology) were added to the samples and incubated at 4°C for 1 hour on a rocker platform. Immunoprecipitates were collected by centrifugation at 1500 rpm for 5 minutes at 4°C while supernatant was discarded. Precipitates were washed three times with 1 ml lysis buffer and centrifuged at 4°C. 75 μl of 2x protein sample buffer was added and the mixture boiled for 10 minutes. Samples were immunoblotted and membranes were probed with mouse anti-GFP (Roche), mouse anti-Ub (Enzo), mouse anti-actin (Santa Cruz Biotechnology), and visualized on a Li-Cor Infrared Imaging System.

### Statistical analysis

Statistical significance was determined by a two-tailed *t* test with significant being P<0.05. Unless otherwise specified, bar graphs represent Mean ± S.E.M. Colocalization analysis was conducted using the Costes method to establish a threshold in Fiji software [41] which also outputs the Mander’s and Pearson’s correlation values. A One-way ANOVA was performed for the series. *** indicates p < 0.0001.

### *Drosophila* strains and genetics

*Drosophila melanogaster* stocks were grown on standard media at 25°C. The *Gal4*/UAS system was used to over-express transgenes [42, 43]. Flip-out clones were made by crossing *hs-Flp[122]; act>y*^+^*>Gal-4 UAS-*GFP virgin females with *UAS*-hairpin RNAi transgenic males from the VDRC (#106679). Larvae were heat shocked at 38°C for 10 minutes. *UAS-Dicer2* VDRC (#60007) was combined with *UAS-*RNAi *genes* to obtain more efficient target gene knock down for all RNAi experiments [44]. The *engrailed-Gal4* (*en*-*Gal4*) flies were obtained from Bloomington (#30564). The MARCM system (mosaic analysis with a repressible cell marker) was used to positively identify clones with RNAi *lbk* expression [45]. Animals were heat shocked at 37°C for one hour after 72 hours of development. Third-instar wing discs were dissected for staining with antibodies to pMad [46].

### Generation of *Drosophila* transgenes

The cDNA of the *Drosophila* type II receptor *punt* was inserted into the fly Gateway vector pTWF (*Drosophila* Genomics Resource Center) and transgenic flies were generated, *P[w*^+^ *UAS-punt-3xFLAG]*. This pTWF vector contains the UASt promoter and P elements end for integration, along with a C-terminal 3xFLAG sequence that was fused in frame to the *punt* cDNA. The *UAS*-RNAi-*lbk* RNAi strain was obtained from Vienna (#106679).

### *Drosophila* antibody staining

Third instar larvae were dissected in chilled 1x Ringers solution. Disc tissue was fixed in formalin (Sigma) for 10 minutes at room temperature. PBST (0.1% Triton X-100 in 1x PBS) was used for the following washing and antibody incubation. Primary antibodies used for staining were rabbit anti-P-MAD (diluted as 1:4000) [46], and mouse monoclonal ANTI-FLAG^®^ M2 antibody (diluted as 1:10000, Sigma). Secondary antibodies, conjugated to Cy3 (diluted 1:200, Jackson ImmunoResearch Lab), and Alexa fluor 633 (diluted 1:100, Invitrogen), were used for detection. All primary antibodies were diluted in PBST and incubated with tissue samples at 4°C overnight. Secondary antibodies were typically incubated with tissue samples for 2 hours at room temperature. Wing imaginal discs were mounted in Vectashield mounting medium (Vector Laboratories) and analyzed using confocal microscopy.

## Results

Our previous work has shown that SMA-10 binds directly to both *C*. *elegans* receptors, SMA-6 and DAF-4 [22]. Importantly, this interaction is conserved in mammals as shown by binding of LRIG1 to both type I and II mammalian BMP receptors [27], but not the ligand. Receptor binding by SMA-10 suggests several possible modes of action of SMA-10, one of which involves trafficking of BMP receptors. In order to investigate the role of SMA-10, we chose to study it in the well characterized, polarized epithelial cells of the *C*. *elegans* intestine, where BMP signaling is required and where there are many cell biological tools available [22, 27, 47].

### SMA-10 colocalizes preferentially with the type I receptor

**Fig 1:**
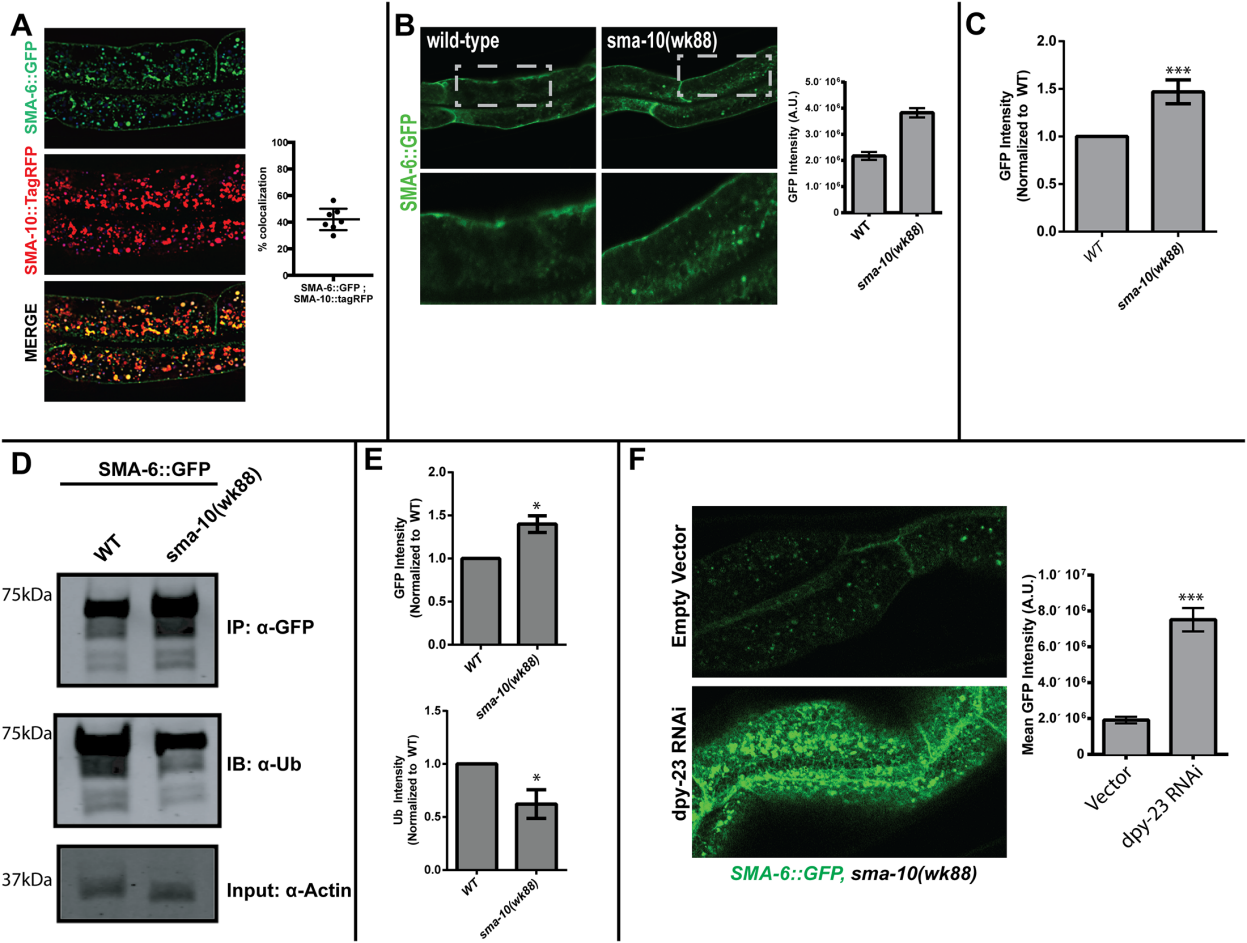
SMA-10 is required for the intracellular trafficking of the type I receptor, SMA*-6*. **(A)** SMA-10 colocalizes preferentially with SMA-6 (type I) intracellularly: SMA-10 shows overlap with both receptors, although the fraction of colocalization with SMA-6 is higher. SMA-6::GFP and SMA-10::tagRFP were expressed in the *C*. *elegans* intestine using the *vha-6* promoter. Young adult animals (L4+24 hrs) were imaged using the confocal microscope and colocalization was performed. Mander’s overlap coefficient for SMA-10::tagRFP and SMA-6::GFP is 0.512. **(B)** Loss of *sma-10* leads to intracellular accumulation of SMA-6. SMA-6 accumulates within intracellular compartments in the *sma-10(wk88)* mutant from three regions from six animals, using a total of 18 data points. **(C)** Total GFP intensity measured from worm lysates from either SMA-6::GFP or SMA-6::GFP,*sma-10(wk88)* mutant background. **(D)** Western blot of ubiquitination state of SMA-6 in wildtype and *sma-10* mutant background. **(E)** Quantification of ubiquitination data, **(F)** Loss of SMA-10 does not affect the biosynthesis or transport of SMA-6 to the plasma membrane. Loss of *dpy-23* prevents internalization of clathrin-dependent cargos. SMA-6 biosynthesis and subsequent trafficking to the plasma membrane is unaffected in *sma-10(wk88)* animals.

Although SMA-10 and its human ortholog LRIG1 physically interact with both receptors, it appears that there is a greater affinity of the two proteins for the type I receptors as compared to the type II receptors [27]. Given this difference, we wanted to determine if SMA-10 preferentially colocalizes with SMA-6 *in vivo*. In order to identify colocalization patterns of SMA-10 with the receptors *in vivo*, we performed confocal microscopy of tagged proteins within the intestine of *C*. *elegans*. Consistent with our biochemical findings [27], we observe colocalization of SMA-10 with both SMA-6 and DAF-4 in sub-cellular compartments (Fig 1A and S1C Fig). Importantly, we show that SMA-10 colocalizes disproportionately with SMA-6 as compared to DAF-4 (42.1% with SMA-6 in Fig 1A and 7.0% with DAF-4 in S1 Fig). These data and our previous biochemical studies of physical interactions suggest [27] that *sma-10* may exert its effects on BMP signaling primarily through regulation of the type I receptor. Given that we have uncovered two separate trafficking pathways for the receptors, these data suggest that SMA-10 may interact or traffic with SMA-6 in its distinct pathway.

### SMA-10 is necessary for intracellular trafficking of SMA-6

To study the effect of loss of *sma-10* on the receptors, we visualized GFP-tagged receptors in wild-type and in *sma-10(wk88)* (Fig 1B), an allele that results in a premature stop codon in the ninth leucine-rich domain [27]. We observe that SMA-6 accumulated intracellularly in *sma-10(wk88*) compared to wild type. A second allele, *sma-10(wk66)* shows the same defects found in *sma-10(wk88)*(data not shown) [27]. We confirmed these accumulations by measuring total GFP fluorescence from worm lysates in the wild-type and *sma-10(wk88)* animals (Fig 1C).

Mammalian LRIG1 is known to regulate EGF by binding to the EGF receptor (EGFR), recruiting the E3 ubiquitin ligase c-Cbl and ubiquitination of EGFR and LRIG1. This leads to trafficking of the receptor (and bound LRIG1) into the lysosome for degradation abolishing further signaling [48]. To determine whether *C*. *elegans* SMA-10 affects ubiquitination of the SMA-6 receptor, we performed an immunoprecipitation of GFP-tagged receptor in the wild-type and *sma-10(wk88)* mutant background (Fig 1D). We observed increased levels of SMA-6 in the *sma-10(wk88)* mutant (Fig 1D, Upper panel) similar to what we observe in our confocal imaging (Fig 1B) and fluorescence measurements (Fig 1C). Further, we show that loss of *sma-10* leads to a significantly decreased ubiquitination signal on SMA-6 (Fig 1D, middle panel). Thus, *sma-10* is required for the maximal ubiquitination of the *C*. *elegans* type I BMP receptor and this ubiquitination may be required for the normal trafficking of SMA-6.

In order to rule out the possibility that SMA-6 accumulations were due to any synthesis and transport to the plasma membrane of the receptors, we examined the accumulation of SMA-6 at the plasma membrane by knocking down *dpy-23*. *dpy-23* is the μ-2 adaptin member of the AP2 complex and is required for the clathrin-dependent endocytosis of various transmembrane receptors [22, 49–51]. Loss of *dpy-23* by RNAi leads to the suppression of clathrin-mediated endocytosis, resulting in accumulation of SMA-6 at the plasma membrane and inhibition of BMP signaling [22]. If *sma-10(wk88)* were to affect the biosynthesis and trafficking of SMA-6 to the plasma membrane, we would expect that SMA-6 would be unable to accumulate at the surface with the depletion of *dpy-23*. We observe that SMA-6 is able to efficiently accumulate at the plasma membrane even in the absence of *sma-10*, suggesting that *sma-10* is required for endocytosis/trafficking once SMA-6 has reached the plasma membrane (Fig 1F).

Taken together, these data suggest that SMA-10 is required for proper endocytosis/trafficking of the receptors via a mechanism that requires ubiquitination of the type I receptor, and loss of *sma-10* leads to receptor accumulation in compartments that are incompatible with signaling.

Having established that *sma-10* is involved with trafficking SMA-6, we asked whether *sma-10* also has a broader role in regulating movement of other cellular receptors. We chose two well-studied cargoes that utilize distinct trafficking pathways to study the effect of *sma-10* on the trafficking of these cargoes—human transferrin receptor (hTfR) and human IL-2 receptor α-chain (hTAC). hTfR enters cells via clathrin-dependent endocytosis while hTAC is internalized in a clathrin-independent manner [16]. We report that the loss of *sma-10* did not alter the localization pattern of these cargoes, although total levels were slightly lower, but not significantly (S2 Fig). EGFR signaling in *C*. *elegans*, mediated by a single EGFR (LET-23), directs several embryonic and larval cell fates and also ovulatory contractions in adult hermaphrodites [52]. We do not observe any of the phenotypes associated with loss of EGF signaling in the *sma-10* animals (data not shown). These data suggest *sma-10* is not a general regulator of receptors, but rather affects a limited number of receptors.

### *Drosophila* LRIG also affects BMP pathways

**Fig 2:**
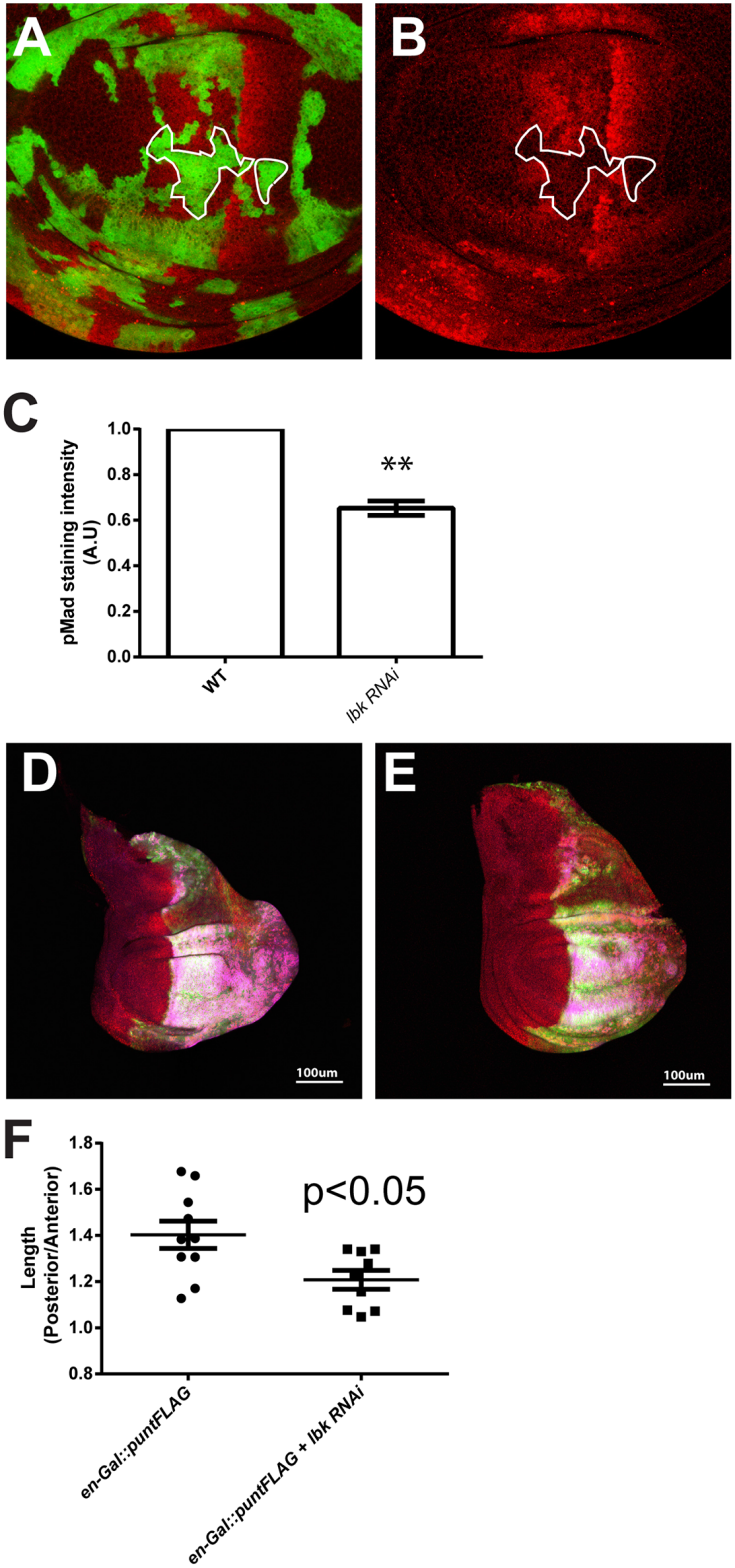
LBK is required for *dpp* signaling in the *Drosophila* wing disc. **(A, B)** Loss of *lbk* results in reduced phosphorylated MAD (pMAD) levels. RNAi-*lbk* clones were made with flip-out technology [45]. **(A)** Green labels clones where lbk is reduced, while red stains for pMad. **(B)** In RNAi-*lbk* clones (green), pMAD (red) was significantly decreased **(C)** compared to the neighboring cells (non-green cells). The genotype of these flies is *hs-Flp[122]/+; act>y*^+^*>Gal-4 UAS-GFP/UAS-*RNAi*-lbk*. **(D)** *punt* (type II receptor) was over expressed in the posterior compartment of the third instar larval wing disc using an *en*-Gal4 driver. The posterior compartment is labeled by GFP expression (green). *punt* expression was labeled by its FLAG tag staining (magenta). The genotype of these flies is *UAS-Dcr2/+; en-*Gal4 *UAS-*GFP*/+; UAS-punt-3xFLAG/+*. **(E)** *punt* was over expressed while the *lbk* gene was knocked down in the posterior compartment of the third instar larval wing disc. The posterior compartment was labeled by GFP expression (green). *punt* expression was labeled by its FLAG tag staining (magenta), and pMad is labeled red. The genotype of these flies is *UAS-Dcr2/+; en-*Gal4 *UAS-*GFP*/ UAS-*RNAi*-lbk; UAS-punt-3xFLAG/+*. Overexpression of *punt* caused the enlargement of posterior compartment, but the effect of *punt* was alleviated by RNAi*-lbk*. **(F)** The size of posterior compartment was significantly diminished. The average ratios of diameters of the anterior to posterior compartments are 1.40 and 1.15 (p<0.05) (S2 Table).

Having established the specificity of *sma-10* regulation of BMP signaling, we wanted to identify if this interaction was conserved in other organisms. We turned to the *Drosophila lbk* gene, an ortholog of *sma-10*. In *Drosophila*, the *decapentaplegic* BMP pathway (*dpp*) is well-studied [53, 54], but the role of *lbk* is not well-defined. Using the MARCM method [45], we made clones expressing *lbk* RNAi in the wing imaginal disc and assayed *dpp* output using the phosophorylated Smad (pMad), a reporter for activation of BMP signaling [46]. In clones expressing *lbk* RNAi, we observed significantly less pMad (Fig 2A and 2B), suggesting that *lbk* is needed for proper signaling of the BMP pathway in *Drosophila*. To determine if a reduction of *lbk* alters signaling strength of the BMP pathway, we over-expressed *punt*, a type II receptor of the *Drosophila* BMP pathway, in the posterior half of the wing imaginal disc using the *en*-*Gal4* driver. Over-expression of many BMP components in the wing disc, including *punt*, cause overgrowth in the wing discs. We would expect depletion of *lbk* to reduce the overgrowth of the wing disk if it were necessary for BMP signaling. When *lbk* RNAi was simultaneously expressed along with *punt* in the posterior compartment of the wing disc, significantly smaller wing discs were observed (p<0.05) (Fig 2D and 2E) (S2 Table). These experiments show that *lbk* is required for optimal signaling of the BMP pathway in *Drosophila*.

### SMA-6 accumulates in the MVB in *sma-10* mutants

Changes in posttranslational modifications of transmembrane receptors have been shown to direct internalization, regulate the sorting of receptors into the MVB, and promote receptor recycling [55]. For example, a ubiquitinated EGFR can be actively internalized into MVBs and degraded, while removing ubiquitin from the receptor at an earlier stage in sorting promotes its recycling [55]. Blocking either the sorting of receptors into MVBs or the recycling of receptors could result in the accumulations we identified of SMA-6 and DAF-4 in *sma-10* mutants.

**Fig 3:**
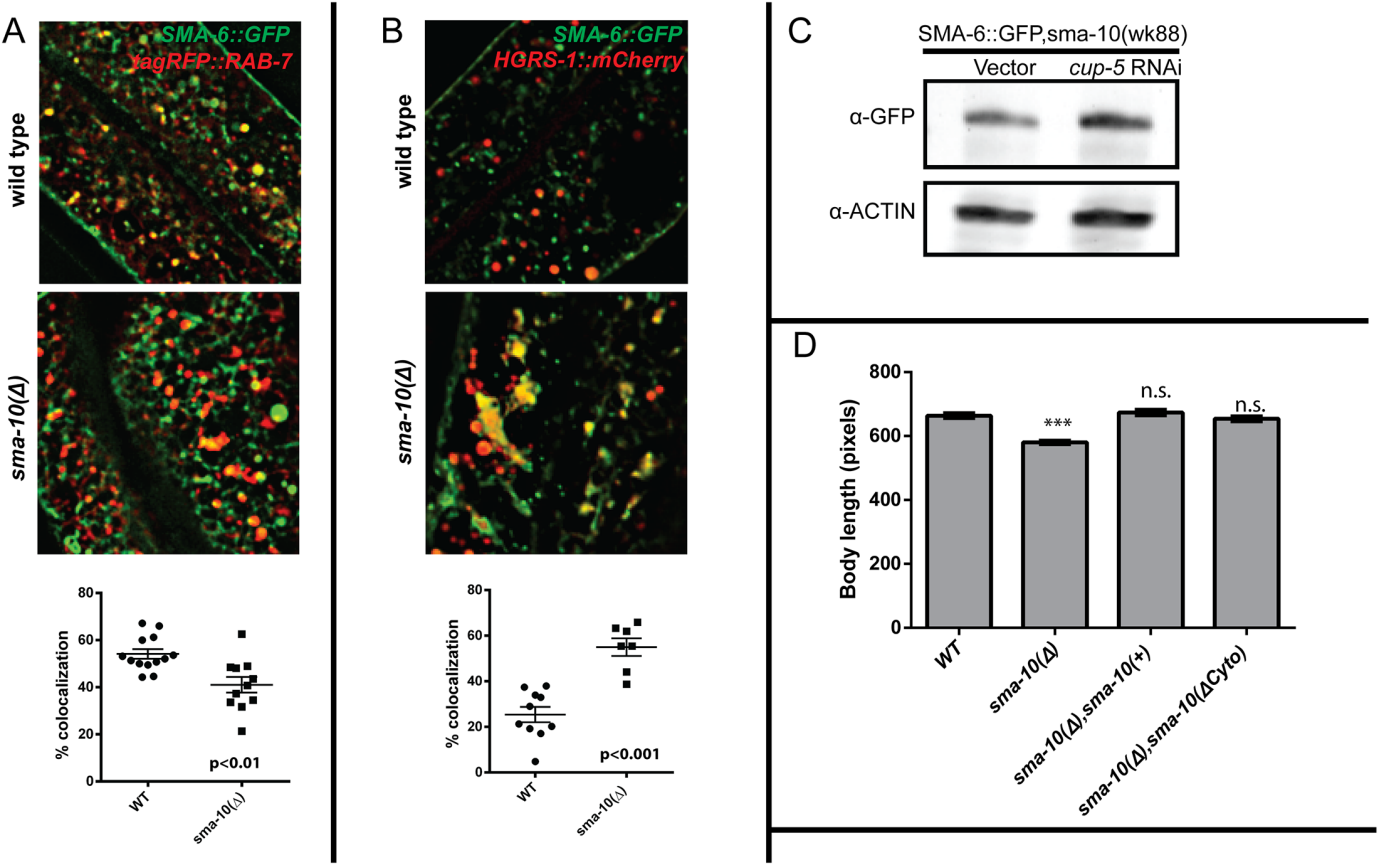
*sma-10* is a critical regulator of SMA-6 trafficking. **(A)** Loss of *sma-10* leads to decreased colocalization of SMA-6 within late endosomes. **(B)** Loss of *sma-10* leads to increased colocalization of SMA-6 within MVBs. **(C)** Mutants of *cup-5*, a necessary component of the lysosome, result in accumulation of cargos destined for the lysosome. *cup-5* RNAi shows accumulation of SMA-6::GFP in a *sma-10* mutant. **(D)** Body size rescue experiments in *C*. *elegans*. The C-terminal cytoplasmic tail of SMA-10 is not required for body size determination in *C*. *elegans*. Plasmid constructs expressing full length *sma-10 [sma-10(+)*,red] and *sma-10* lacking the cytoplasmic tail [*sma-10(Δcyto)*,blue] from a hypodermal-specific promoter were injected into the *sma-10(wk88)* mutant background. At least 30 transformed animals were assayed for body size at L4 + 24 hr for each genotype. As observed, full length *sma-10* (red) as well as *sma-10* lacking the cytoplasmic tail (blue) fully rescue the small body size of *sma-10(wk88)* indicating that the cytoplasmic tail is not required for the rescue of the body size defects. Bar graphs represent Mean ± S.E.M. A One-way ANOVA was performed for the series. *** indicates p < 0.0001 as compared to the wild-type. A minimum of 15 animals per line were assayed for body size.

Our previous work has shown that SMA-6 is internalized through clathrin-dependent endocytosis, and is recycled back to the surface via the retromer complex [22]. Loss of retromer-mediated trafficking leads to degradation of cargo via the lysosome [56–59]. Since we observe intracellular accumulation of SMA-6 in the *sma-10* mutant animal (Fig 1), it is unlikely that *sma-10* affects retromer-mediated trafficking of the type I receptor. Identifying the endosomal compartments where the type I receptor normally traffics and pinpointing compartments where it accumulates in the *sma-10(wk88)* animal, will allow us to define the role of *sma-10* in regulation of receptor trafficking. In order to determine which intracellular compartments SMA-6 traffics to and how this changes with the loss of *sma-10*, we performed colocalization experiments of SMA-6 with various endosomal markers [20, 60] in wild-type and in *sma-10* mutants (Fig 3 and S3 Fig).

We first demonstrated that in wild-type animals SMA-6 colocalizes to several compartments in the endocytic trafficking pathway, including early (tagRFP::RAB-5) and late endosomes (tagRFP::RAB-7), recycling endosomes (RME-1::tagRFP), MVBs (HGRS-1::mCherry) and lysosomes (LMP-1::tagRFP) (Fig 3A, 3B and S3 Fig). We then compared the change, if any, in the colocalization pattern of SMA-6 in the *sma-10(wk88)* animal to identify the sites at which SMA-6 is mis-localized. We observe that loss of *sma-10* leads to a significantly reduced colocalization of SMA-6 within late endosomes (Fig 3A) and an increase in the colocalization with MVBs (Fig 3B). MVBs contain cargo that can be recycled, or once recruited into intraluminal vesicles, are destined for degradation through subsequent fusion to the lysosome [14, 16]. Accumulation within the MVB suggests that at least some of the mis-trafficked SMA-6 may be degraded within the lysosome. Indeed, when lysosome function is blocked using RNAi against *cup-5*, a gene necessary for lysosome function, we see an increased accumulation of SMA-6 in the *sma-10* background (Fig 3C) [47].

The mammalian homolog, LRIG1, recruits the E3-ubiquitin ligase, c-Cbl through interaction with regions in LRIG1’s cytoplasmic tail. Compared to the mammalian LRIG1 protein, *C*. *elegans* SMA-10 has a very short cytoplasmic tail (20 amino acids compared to approximately 280 amino acids in mammals), and we were interested to identify whether it had a role in signaling. Intriguingly, the 20 amino acids present in the cytoplasmic tail domain of SMA-10 show a limited homology with a region in the LRIG1 cytoplasmic tail necessary for the interaction with an E3 ubiquitin-protein ligase, c-Cbl (S4 Fig) (These sequences are missing in LRIG2 and LRIG3). We used the rescue of the small body size of the *sma-10(wk88)* mutant animal as a functional readout for activity. As expected, we observe a complete rescue when the full length genomic *sma-10* was injected into the *sma-10(wk88)* mutant animal (Fig 3D). However, we also observe a complete rescue of the body size for three different transgenic lines expressing a truncated version of *sma-10* that lacks the cytoplasmic tail (Fig 3D). These data suggest that, unlike the mammalian LRIG1 cytoplasmic tail’s requirement for EGF regulation, nematode *sma-10* does not require the cytoplasmic tail for regulating BMP signaling.

### Subcellular localization of SMA-10 suggests early stages of trafficking

**Fig 4:**
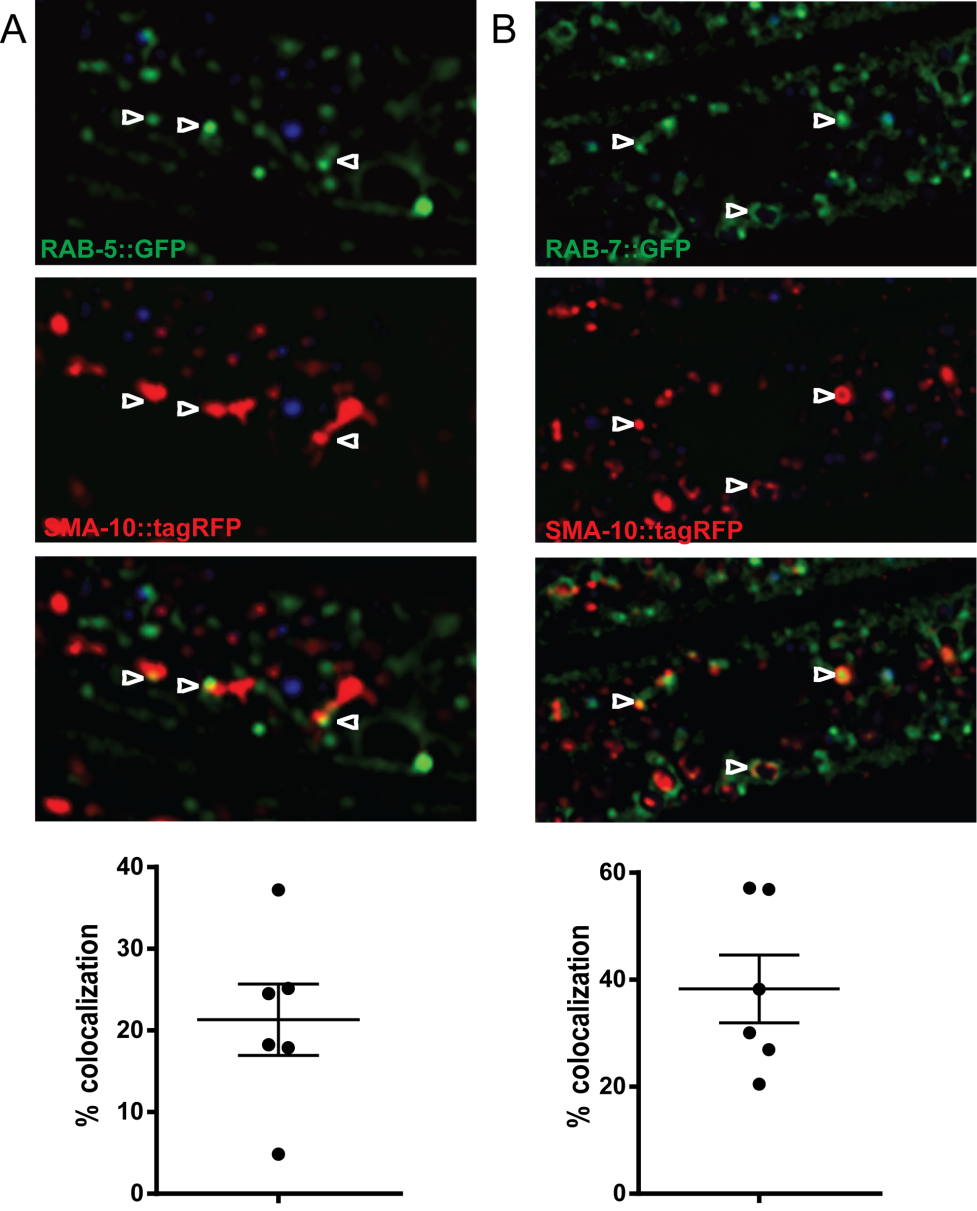
SMA-10 colocalizes at the surface, early and late endosomes. SMA-10::tagRFP was co-expressed with early endosomal marker, RAB-5::GFP **(A)**, late endosomal marker RAB-7 **(B**). Images were taken at the L4 + 24 hr stage using a confocal microscope and colocalization was performed.

Given that loss of *sma-10* leads to the aberrant accumulation of SMA-6 within the MVB, it suggests that SMA-10 acts either upstream of or at the MVB. In order to determine the cellular localization patterns of SMA-10, we performed a series of colocalization studies using TagRFP-tagged SMA-10 and a set of GFP-tagged endosomal markers (Fig 4).

The presence of a signal peptide and transmembrane domain in SMA-10 suggests that it is present at the plasma membrane, at least transiently. SMA-6 requires an RME-1 to recycle back to the plasma membrane, and we first tested whether SMA-10 localized with the recycling endosome marker, RME-1 [22]. We did not find significant overlap of the two markers, suggesting that SMA-10 does not mediate recycling of SMA-6 through recycling endosomes (data not shown). To test if SMA-10 localizes to the early and late endosomes, we tested colocalization of SMA-10::TagRFP to early endosome marker GFP::RAB-5 and to late endosome marker GFP::RAB-7 (Fig 4A and 4B). Significant colocalization of SMA-10 was identified with both the early and late endosomes. Its cell surface localization and presence at early endosomes suggests an early function for SMA-10. The significant late accumulation in late endosomes could indicate a late function or simply a consequence of its early function.

## Discussion

Our previous work has identified *C*. *elegans sma-10* as a conserved positive regulator of BMP signaling and shown that both worm,SMA-10, and human, LRIG1, bind to their receptors respectively [27], with more binding to the type I receptor compared to the type II receptor. In our current study, we show that nematode SMA-10 preferentially colocalizes with the type I receptor SMA-6 (Fig 1A). These results suggest that the accumulations of SMA-6 we observe in the *sma-10(wk88)* mutant may be a direct consequence of loss of *sma-10*, whereas the trafficking defect in the type II receptor DAF-4 may be indirect. The binding of SMA-10 to SMA-6 suggests a limited number of mechanisms of action. Another type I receptor, DAF-1, functions with DAF-4 in the dauer pathway [61]. We have not assessed whether SMA-10 affects DAF-1. In vertebrates, LRIGs have been shown to affect receptors through different mechanisms. LRIGs enhance EGF receptor degradation through interactions with c-Cbl [48], alter recruitment of GDNF receptors to lipid rafts [62], inhibit ligand-receptor interactions [63, 64], or regulate receptor shedding [65].

Clues to the molecular function of *sma-10* come from observations of altered ubiquitination levels in SMA-6 when SMA-10 is absent. We observe reduced ubiquitination in SMA-6 when SMA-10 is absent (Fig 1D and 1E), increased colocalization of SMA-6 within the MVB (Fig 3B), and the overall accumulation of SMA-6 (Fig 1). Ubiquitin networks regulate endocytic trafficking and/or degradation of a growing number of receptors [66]. In addition, many E3 ligases contain transmembrane (TM) domains to physically tether the ubiquitin complex to specific organelles to compartmentalize the ligase activity of these enzymes. For some E3 ligases that do not contain this TM domain, scaffold proteins exist to restrict their activity to specific organelles [67]. SMA-10 may function as such a transmembrane scaffolding protein. SMA-10 regulation of SMA-6 localization may be through ubiquitination-mediated sorting into the MVB. Alternatively, the loss of ubiquitination of SMA-6 could be a downstream effect of the primary function of SMA-10. LRIG1, the mammalian ortholog of SMA-10, regulates EGF signaling by first binding to the EGFR and then recruiting the E3-Ubiquitin ligase, c-Cbl through interactions with its C-terminus. c-Cbl then ubiquitinates both LRIG1 and EGFR, leading to their degradation within the lysosome. The short cytoplasmic tail is dispensable for body size determination in *C*. *elegans* (Fig 3E) as well as for stimulation of BMP signaling in mammalian cells [27]. However, since there is a short stretch of homology of the *C*. *elegans* cytoplasmic tail sequences with human Lrig1 and *Drosophila lbk* (S4 Fig), this does not preclude the importance of the tail for other functions.

Since SMA-10 is present at the surface with SMA-6, it is possible that SMA-10 may be required to bring the receptors in proximity or to protect surface proteins from modification. In *C*. *elegans*, we know that the clathrin-dependent pathway is the major entry for the type I receptor. Blocking this pathway with *dpy-23* (a key component of the AP2 adapter complex), we do not find surface accumulation of the type II receptor [22], suggesting its entry through non-clathrin pathways. Since signaling occurs when the receptors come together, it is necessary for the different rafts carrying the type I and II receptors to merge during the early phase of signal transduction. Multiple examples exist in which molecules are known to bring together microdomains. A third mammalian TGFβ receptor, TβRIII, has been shown to direct the type I and type II receptors into clathrin-dependent endocytosis and to increase trafficking of the type II receptor into early endosomal compartments, thereby increasing TGFβ signaling [68]. Likewise, betulinic acid induces translocation of receptors from lipid-raft/caveolae-mediated domains to non-caveolae domains, resulting in a rapid increase in reporter gene activation [69]. The opposite effect is seen with Euphol, which induces degradation of the receptors by causing the receptors to move into lipid-raft microdomains [69]. Tetraspanin-enriched microdomains are important for BMP signaling and may also participate in bringing signaling molecules together [70].

These data support the idea that the placement of receptors in plasma surface microdomains strongly influences signaling strength. We have previously shown that SMA-10 binds to both receptors [27] and that each receptor recycles back to the surface through distinct pathways [22]. Inappropriate fusion of their raft microdomains at the surface may lead to intracellular trafficking problems and aberrant accumulations in their distinct routes of trafficking. In *sma-10* animals, we observe that the two receptors accumulate in distinct intracellular compartments (Figs 1 and 3, data not shown for DAF-4). Given that SMA-6 is unaffected by *arf-6* (a key regulator of the endocytic recycling compartment to plasma membrane) mutants, while DAF-4 accumulates [22], it suggests that the two receptors have not separated at the stage where SMA-10 acts, possibly an earlier step of trafficking. This is consistent with a possible function at the surface for bringing the microdomains together.

Regulation of BMP signaling can also occur via a process called ectodomain shedding [34, 71, 72]. Recently, LRIG2 was shown to inhibit ectodomain shedding of neogenin [65], a gene necessary for BMP signaling. Given that several LRIG binding partners are also ADAM substrates, such as ErbB4, this has led to the suggestion that an additional function of Lrig is to prevent ectodomain shedding of bound proteins [65]. It is interesting to note that some members of the TGFβ family undergo ectodomain shedding and known membrane-associated proteases regulate signaling [34, 71, 72]. Consistent with this, in *C*. *elegans*, neogenin (UNC-40), a positive regulator of BMP signaling, undergoes ectodomain shedding by an ADAM protease (SUP-17) [73]. It was hypothesized that tetraspanins in the worm protect UNC-40 cleavage by SUP-17 [73] and thereby promote BMP signaling. It is possible that SMA-10 performs a role similar to that of LRIG2 by protecting one or more of the surface proteins necessary for efficient BMP signaling from ectodomain shedding. DRAG-1 is also part of the BMP cell surface complex in *C*. *elegans* [74]. However, it is unlikely to be involved in the core SMA-10 function, since it is missing from *Drosophila* (but present in mammals), where we show that the SMA-10 ortholog (*lbk*) affects BMP signaling.

The human homolog of SMA-10, LRIG1, negatively regulates the epidermal growth factor (EGF) signaling (Gur et al 2005). The *C*. *elegans* EGFR, *let-23*, is responsible for vulval development, and perturbations in the pathway lead to distinct vulval developmental phenotypes [75]. A loss-of-function phenotype leads to an animal that is vulvaless (Vuv) while a gain-of-function mutation leads to an animal that is multivulva (Muv) [75]. If SMA-10 is involved in the regulation of EGF signaling in the worm, we would expect that a loss-of-function allele of *sma-10* would lead to an animal that is Vuv. We do not observe any obvious vulval defects in the *sma-10* mutant strain (data not shown). Based on these observations, *sma-10*, at least in the nematode, does not affect the EGF signaling pathway, or if so, the effects are minimal or too subtle to be observed without a sensitized background [76–78]. Further, in order to rule out that *sma-10* is a general regulator of trafficking, we asked whether SMA-10 also regulated other receptors in *C*. *elegans*. The human transferrin receptor (hTfr) is a cargo that has been shown to traffic via a clathrin-dependent mechanism, while the α-chain of the human IL-2 receptor TAC (hTAC) traffics via a clathrin-independent mechanism [16]. We find that SMA-10 does not significantly alter the trafficking of these receptors (S3 Fig) and thus likely functions to regulate BMP, and possibly a small group of receptors.

Our work shows that the function of *sma-10* as a positive regulator of BMP signaling is conserved in other phyla. We show that pMad levels are altered in *Drosophila lbk* mutants (Fig 2) and have previously shown that the *Drosophila* ortholog can rescue *C*. *elegans sma-10* mutants [27]. Additionally, we have shown that expression of SMA-10 in mammalian cells stimulates BMP signaling [27]. Our current work expands on the function of this conserved protein and identifies its role as a critical regulator of trafficking and ubiquitination of the type I receptor in *C*. *elegans*. Whether these phenotypes are a direct or indirect consequence of SMA-10 function is currently being investigated.

## Supporting information

S1 Table *C*. *elegans* strains used in this study. (DOCX)

S2 Table Measurement data for *punt* discs. (DOCX)

S1 Fig DAF-4 accumulates in *sma-10* mutants. **(A)** Loss of *sma-10* leads to intracellular accumulation of DAF-4. **(B)** Total GFP intensity measured from worm lysates from either DAF-4::GFP or DAF-4::GFP,*sma-10(wk88)* mutant background. **(C)** The colocalization between DAF-4 and SMA-10 is minimal.
(TIF)

S2 Fig *sma-10* is not a general regulator of receptor trafficking. **(A)**. hTAC::GFP and hTfr::GFP [16] were expressed in the intestinal cells of wild-type and *sma-10* mutant animals. Fluorescence confocal microscopy was performed and intensity measured using Fiji software in the same manner as in Fig 1. As observed, there is no significant decrease in the total fluorescence of GFP in the *sma-10* animals. Further, there is not gross change in the localization patterns of the GFP-tagged cargos. **(B)** Western blots of animals from **(A)**.
(TIF)

S3 Fig Distribution of SMA-6 is not affected by loss of *sma-10*. Colocalization data in early endosomes **(A)**, recycling endosomes **(B)**, or lysosomes **(C**) as detected by the colocalization of the endocytic markers tagRFP::RAB-5, RME-1::tagRFP and LMP-1::tagRFP with SMA-6::GFP respectively.
(TIF)

S4 Fig A small domain on the SMA-10 C-terminal tail exhibits modest conservation with fly and human orthologs. **(A)** The cytoplasmic tails vary greatly in length among the three organisms. Green boxes indicate the regions which show most conservation with the small 20 aa tail of *C*. *elegans*. **(B)** Alignments of the amino acids from the green region from panel A.
(TIF)
