# Supplementary material for: C. elegans SMA-10 regulates BMP receptor trafficking
Source: PLoS One. 2017 Jul 13;12(7):e0180681. doi: 10.1371/journal.pone.0180681 (PMC5509155; doi:10.1371/journal.pone.0180681)
Supplement: S1 Table — (DOCX) [file pone.0180681.s001.docx]

| **STRAIN NAME** | **Genotype** |
| --- | --- |
| LT944 | *sma-6::GFP,tagRFP::rab-5* |
| LT970 | *sma-6::GFP,tagRFP::rab-5,sma-10(wk88)* |
| LT915 | *sma-10::tagRFP,GFP::rab-7* |
| LT951 | *sma-6::GFP, tagRFP::rab-7* |
| LT965 | *sma-6::GFP, tagRFP::rab-7,sma-10(wk88)* |
| LT910 | *sma-10::tagRFP,MANS::GFP* |
| LT859 | *sma-6::GFP,lmp-1::tagRFP, sma-10(wk88)* |
| LT886 | *sma-6::GFP,lmp-1::tagRFP* |
| LT856 | *sma-6::GFP,hgrs-1::mCherry,sma-10(wk88)* |
| LT894 | *sma-6::GFP,hgrs-1::mCherry* |
| LT913 | *sma-10::tagRFP,rme-1::GFP* |
| LT943 | *sma-6::GFP,rme-1::tagRFP* |
| LT947 | *sma-6::GFP,rme-1::tagRFP,sma-10(wk88)* |
| RT2496 | *sma-6::GFP* |
| LT821 | *sma-6::GFP,sma-10(wk88)* |
