# Supplementary material for: C. elegans SMA-10 regulates BMP receptor trafficking
Source: PLoS One. 2017 Jul 13;12(7):e0180681. doi: 10.1371/journal.pone.0180681 (PMC5509155; doi:10.1371/journal.pone.0180681)
Supplement: S2 Table — (DOCX) [file pone.0180681.s002.docx]

| Data for Fig 2D.  enGAL4> UAS-puntFLAG | |  |  |  |
| --- | --- | --- | --- | --- |
| wing discs | **P (μm)** | **A (μm)** | **P/A** |  |
| disc1 | 260.170 | 220.822 | 1.170103 |  |
| disc2 | 288.370 | 174.955 | 1.305893 |  |
| disc3 | 270.112 | 187.889 | 1.543894 |  |
| disc4 | 245.662 | 198.534 | 1.307485 |  |
| disc5 | 332.971 | 196.529 | 1.677148 |  |
| disc6 | 272.603 | 182.095 | 1.387088 |  |
| disc7 | 268.210 | 160.385 | 1.472912 |  |
| disc8 | 266.005 | 171.443 | 1.658540 |  |
| disc9 | 237.020 | 226.222 | 1.382500 |  |
| disc10 | 254.921 | 220.822 | 1.126862 |  |

| **AVG P/A** |
| --- |
| 1.38 |

| Data for Fig 2E.  enGAL4> UAS-puntFLAG + RNAi-lbk | | |  |  |
| --- | --- | --- | --- | --- |
| wing discs | **P (μm)** | **A (μm)** | **P/A** |  |
| disc1 | 205.162 | 219.570 | 1.228383 |  |
| disc2 | 235.325 | 193.382 | 1.071754 |  |
| disc3 | 247.277 | 179.860 | 1.278697 |  |
| disc4 | 241.055 | 230.777 | 1.340237 |  |
| disc5 | 266.875 | 193.559 | 1.156419 |  |
| disc6 | 202.678 | 217.429 | 1.047112 |  |
| disc7 | 289.343 | 224.212 | 1.330747 |  |
| disc8 | 300.561 | 193.980 | 1.340521 |  |
| disc9 | 208.749 | 219.570 | 1.076137 |  |

| **AVG P/A** |
| --- |
| 1.15 |

Unpaired t-test: p<0.05 for AVG P/A
