# Supplementary figures and images for: C. elegans SMA-10 regulates BMP receptor trafficking
Source: PLoS One. 2017 Jul 13;12(7):e0180681. doi: 10.1371/journal.pone.0180681 (PMC5509155; doi:10.1371/journal.pone.0180681)

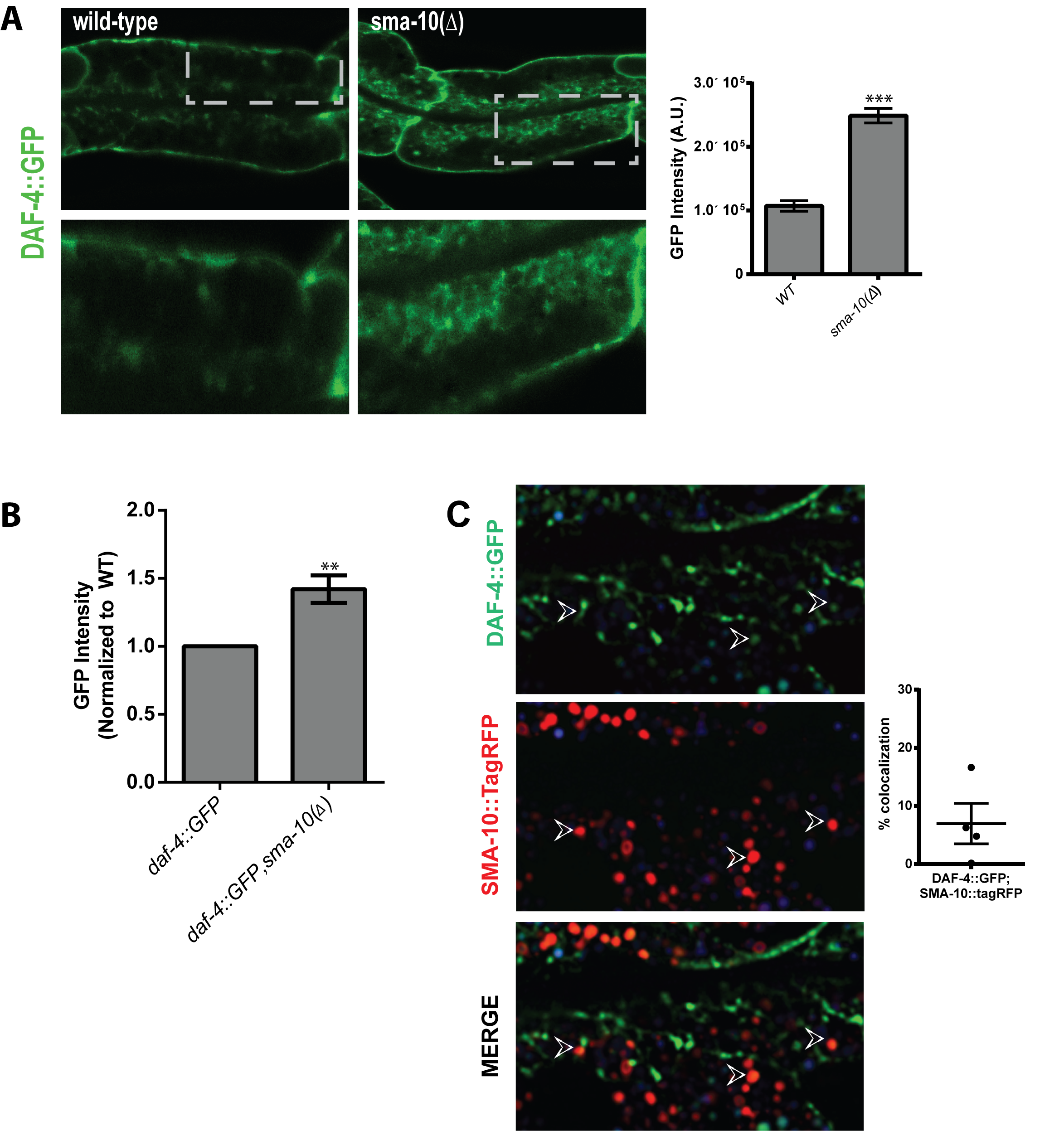

Supplement: S1 Fig — (A) Loss of sma-10 leads to intracellular accumulation of DAF-4. (B) Total GFP intensity measured from worm lysates from either DAF-4::GFP or DAF-4::GFP,sma-10(wk88) mutant background. (C) The colocalization between DAF-4 and SMA-10 is minimal. (TIF) [file pone.0180681.s003.tif]

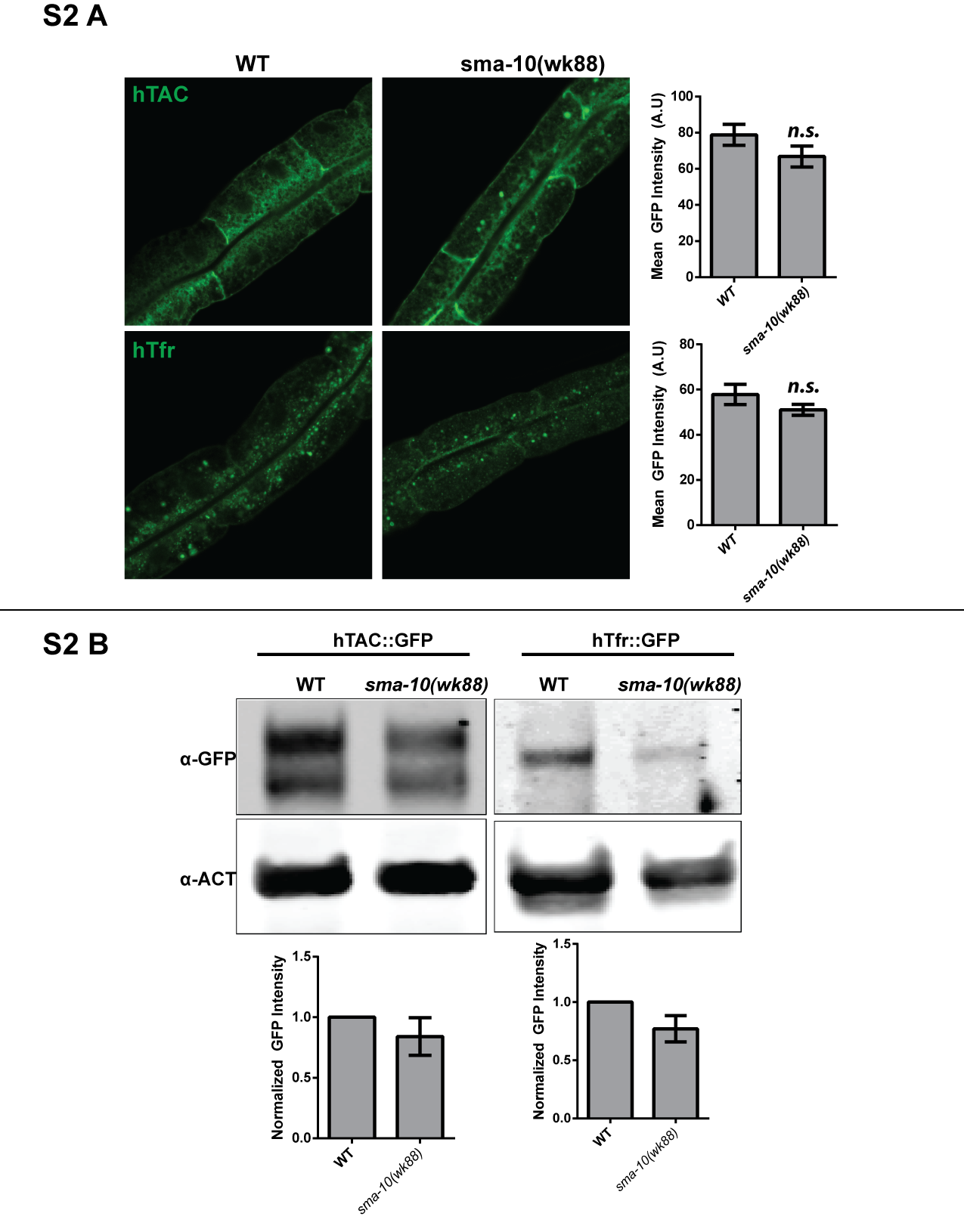

Supplement: S2 Fig — (A). hTAC::GFP and hTfr::GFP [16] were expressed in the intestinal cells of wild-type and sma-10 mutant animals. Fluorescence confocal microscopy was performed and intensity measured using Fiji software in the same manner as in Fig 1. As observed, there is no significant decrease in the total fluorescence of GFP in the sma-10 animals. Further, there is not gross change in the localization patterns of the GFP-tagged cargos. (B) Western blots of animals from (A). (TIF) [file pone.0180681.s004.tif]

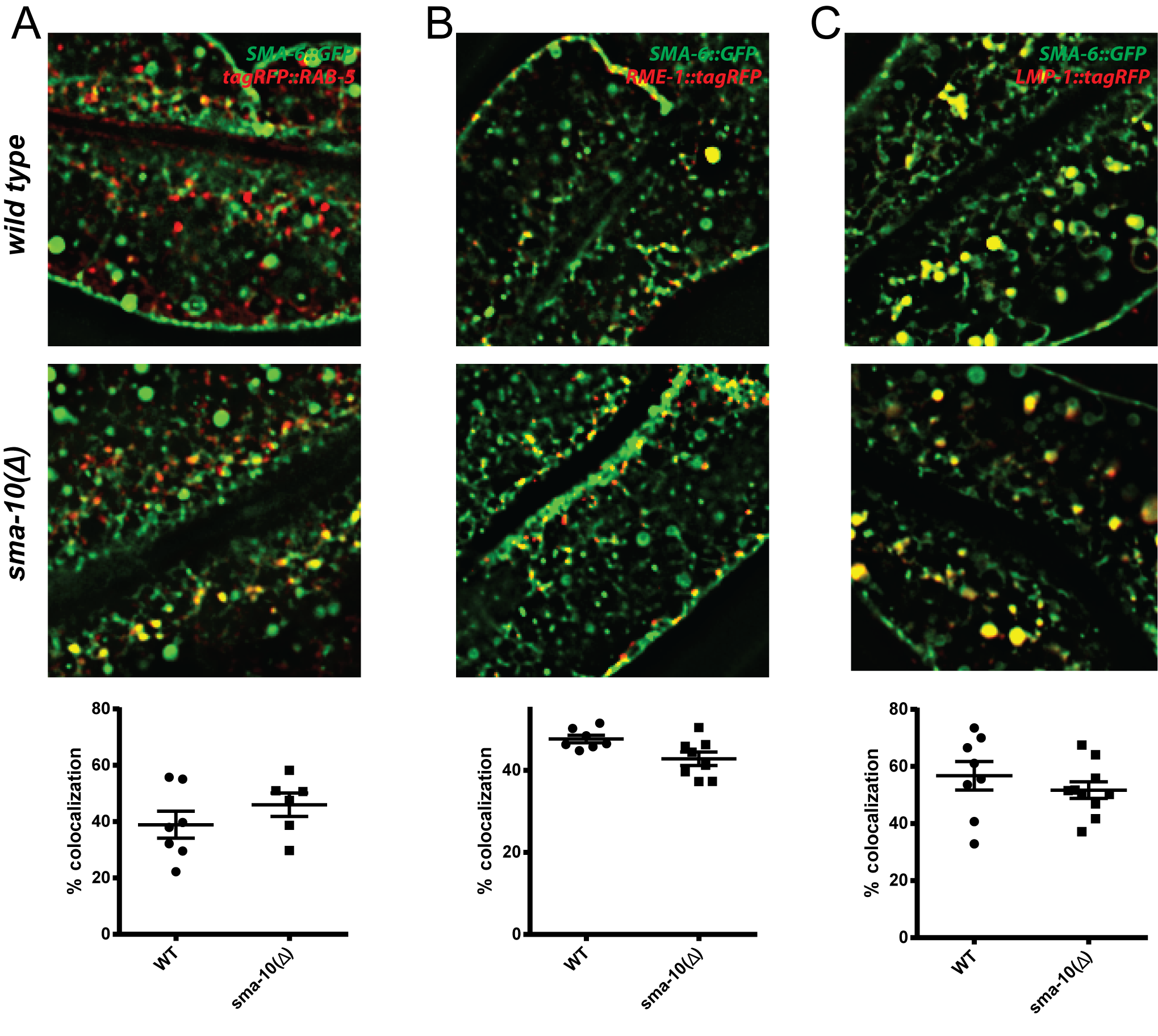

Supplement: S3 Fig — Colocalization data in early endosomes (A), recycling endosomes (B), or lysosomes (C) as detected by the colocalization of the endocytic markers tagRFP::RAB-5, RME-1::tagRFP and LMP-1::tagRFP with SMA-6::GFP respectively. (TIF) [file pone.0180681.s005.tif]

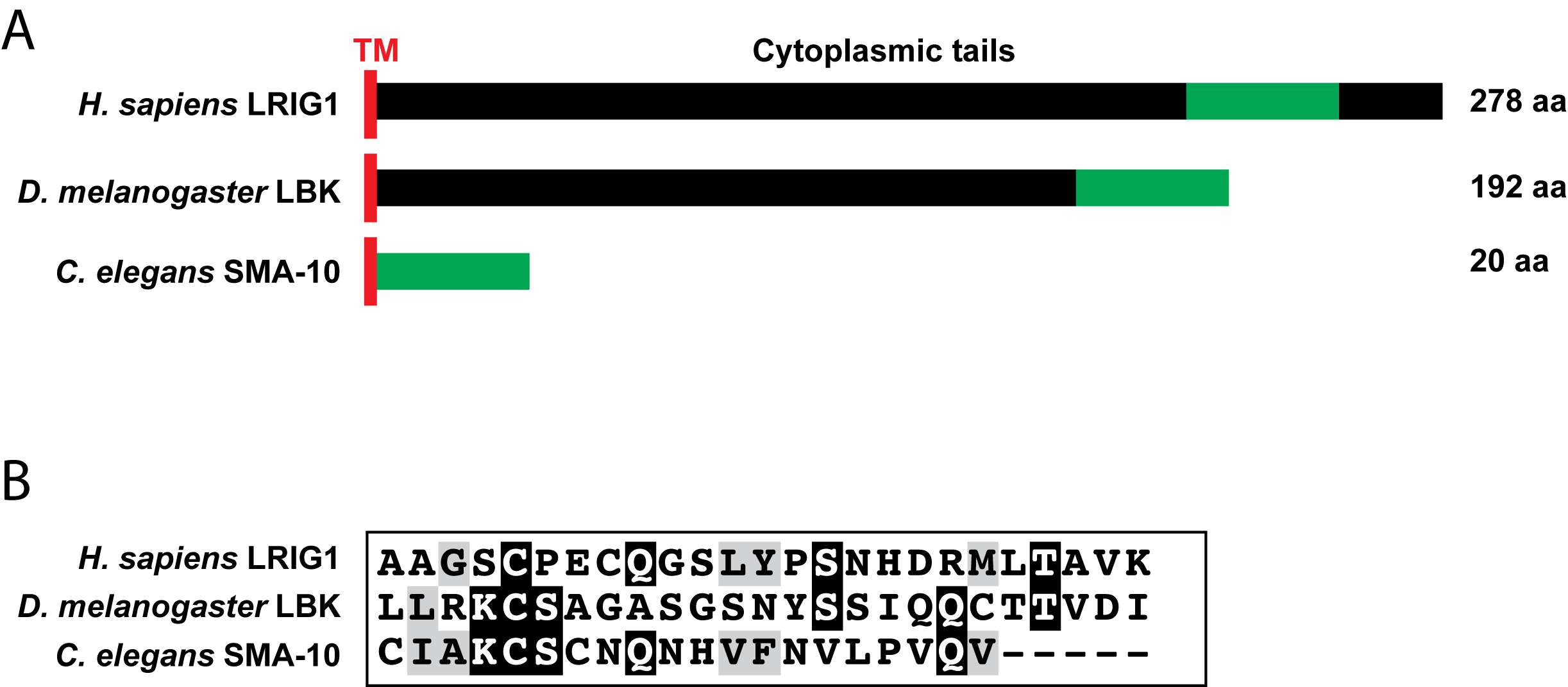

Supplement: S4 Fig — (A) The cytoplasmic tails vary greatly in length among the three organisms. Green boxes indicate the regions which show most conservation with the small 20 aa tail of C. elegans. (B) Alignments of the amino acids from the green region from panel A. (TIF) [file pone.0180681.s006.tif]
